# High-dynamic-range coherent diffractive imaging: ptychography using the mixed-mode pixel array detector

**DOI:** 10.1107/S1600577514013411

**Published:** 2014-08-07

**Authors:** Klaus Giewekemeyer, Hugh T. Philipp, Robin N. Wilke, Andrew Aquila, Markus Osterhoff, Mark W. Tate, Katherine S. Shanks, Alexey V. Zozulya, Tim Salditt, Sol M. Gruner, Adrian P. Mancuso

**Affiliations:** a European XFEL GmbH, Hamburg, Germany; b Department of Physics, Cornell University, Ithaca, NY, USA; c Institut für Röntgenphysik, Georg-August-Universität Göttingen, Göttingen, Germany; d Deutsches Elektronen-Synchrotron DESY, Hamburg, Germany; e Cornell High Energy Synchrotron Source (CHESS), Cornell University, Ithaca, NY, USA; f Kavli Institute of Cornell for Nanoscience, Ithaca, NY, USA

**Keywords:** pixel array detectors, coherent X-ray diffractive imaging, ptychography

## Abstract

Coherent (X-ray) diffractive imaging (CDI) is an increasingly popular form of X-ray microscopy, mainly due to its potential to produce high-resolution images and the lack of an objective lens between the sample and its corresponding imaging detector. One challenge, however, is that very high dynamic range diffraction data must be collected to produce both quantitative and high-resolution images. In this work, hard X-ray ptychographic coherent diffractive imaging has been performed at the P10 beamline of the PETRA III synchrotron to demonstrate the potential of a very wide dynamic range imaging X-ray detector (the Mixed-Mode Pixel Array Detector, or MM-PAD). The detector is capable of single photon detection, detecting fluxes exceeding 1 × 10^8^ 8-keV photons pixel^−1^ s^−1^, and framing at 1 kHz. A ptychographic reconstruction was performed using a peak focal intensity on the order of 1 × 10^10^ photons µm^−2^ s^−1^ within an area of approximately 325 nm × 603 nm. This was done without need of a beam stop and with a very modest attenuation, while ‘still’ images of the empty beam far-field intensity were recorded without any attenuation. The treatment of the detector frames and CDI methodology for reconstruction of non-sensitive detector regions, partially also extending the active detector area, are described.

## Abstract 2

The advantages of a novel wide dynamic range hard X-ray detector are demonstrated for (ptychographic) coherent X-ray diffractive imaging.

## 1. Introduction   

X-ray coherent diffractive imaging (CDI) is a promising and increasingly popular technique for the structure determination of non-periodic organic and inorganic materials (Nugent, 2010 ▶; Quiney, 2010 ▶; Thibault & Elser, 2010 ▶). The chief attraction of CDI is its lensless nature as an imaging method, requiring no optic between the sample and detector. A consequence of avoiding an image-forming optic, however, is the need to measure diffraction patterns produced from the sample, rather than direct (real-space) images. For the majority of CDI variants, *i.e.* whenever diffraction from the sample is recorded in the optical far-field, these patterns exhibit a very large dynamic range to be detected (Nugent, 2010 ▶; Quiney, 2010 ▶). Ideally, this involves simultaneously measuring (i) the intensity of the direct beam, (ii) high-intensity diffraction at near-zero scattering angles that correspond to low-resolution information, and (iii) low-intensity diffraction at the wider angles that correspond to high-resolution information. Since the highest-intensity central part should always be measured to preserve the information related to the quantitative low-resolution information of the object (Thibault *et al.*, 2006 ▶), an increase in resolution is generally accompanied by an increase in the required dynamic range of the detection device.

Furthermore, in contrast to plane-wave CDI (Miao *et al.*, 1999 ▶; Chapman *et al.*, 2006 ▶), more robust and now most-widely used implementations of CDI rely on a structured non-planar illumination and multiple measurements (Williams *et al.*, 2006 ▶; Putkunz *et al.*, 2011 ▶; Rodenburg *et al.*, 2007 ▶; Thibault *et al.*, 2008 ▶; Schropp *et al.*, 2012 ▶), requiring nanometer-scale mechanical stability of the experimental set-up during the measurement process (Takahashi *et al.*, 2011 ▶).

Maximizing the coherent flux incident on a sample often decreases the required measurement time, and is thus a prerequisite to reduce the detrimental effects of mechanical instabilities in an experimental set-up and hence to optimize the achievable resolution. As a consequence, however, detection of the experimental diffraction patterns becomes increasingly challenging. This becomes even more important in view of current and future ultrahigh-brightness storage rings (Bei *et al.*, 2010 ▶) and energy-recovery linacs (Bilderback *et al.*, 2010 ▶; Hoffstaetter *et al.*, 2013 ▶). Therefore, the detection of high-dynamic-range diffraction patterns is one of the most significant limitations to the progress and wider applicability of CDI today. Here we report on the use of a new type of X-ray detector that addresses this problem.

The experiments described below were performed at the P10 beamline of the PETRA III synchrotron source at DESY (Hamburg, Germany) using exposures of 0.1 s duration; this foreshadows exposures of a few milliseconds long that will be feasible at planned, even brighter, X-ray sources. Hence, it is desirable that the detector can read out an exposure in less than a millisecond, that is, to frame at kHz rates. This already excludes most CCD detectors (which also suffer from a limited single-frame dynamic range).

Photon-counting detectors are being designed to meet this frame-rate specification (Broennimann *et al.*, 2006 ▶), but typically start to suffer significant count-rate dead-time losses at ∼10^6^ X-rays pixel^−1^ s^−1^ (Trueb *et al.*, 2012 ▶), an intensity that is easily exceeded at low diffraction angles even at existing synchrotron X-ray facilities. Two approaches have been used to mitigate this problem. (i) Diffusors can be inserted upstream of the sample to amplify high spatial frequencies in the diffraction pattern and decrease the dynamic range required of the detectors (Maiden *et al.*, 2013 ▶). This introduces experimental complications of an added element in the beam and reduced flux due to attenuation (although most of the effect is primarily on the phase). (ii) Alternatively, a semi-transparent beam stop may be inserted in front of the detector to reduce the intensity at low diffraction angles (Wilke *et al.*, 2013 ▶). This, however, introduces the complication of an additional element that has to be tailored in thickness, material and/or size for different experiments and samples.

### 1.1. Mixed-mode pixel array detector   

Ideally, it is desirable to use a detector that (*a*) can frame at kHz rates, (*b*) does not suffer count-rate limitations within the high fluence parts of a diffraction pattern, yet (*c*) is still able to efficiently detect single photons for the low fluence parts of the pattern. In this article we describe experiences with the high-dynamic-range Mixed-Mode Pixel Array Detector (MM-PAD) (Angello *et al.*, 2004 ▶; Vernon *et al.*, 2007 ▶; Tate *et al.*, 2013 ▶) that meets these three specifications and is used for a high-flux CDI experiment, without the need for beam stops or phase diffusors. Although the MM-PAD as presently configured was not designed for use at X-ray free-electron lasers (XFELs), needs at XFELs have since catalyzed the development of several detectors specifically designed for XFEL use, including the Cornell SLAC PAD (Philipp *et al.*, 2007 ▶, 2010 ▶), the Adaptive Gain Integrating Pixel Detector (AGIPD) (Henrich *et al.*, 2011 ▶) and the Large Pixel Detector (LPD) (Blue *et al.*, 2009 ▶). In this regard an excellent history of hybrid X-ray pixel array detectors (PADs) may be found within Graafsma (2010 ▶).

The MM-PAD^1^ uses a 500 µm-thick fully depleted silicon sensor to absorb X-rays and create mobile charge carriers. These charge carriers are conveyed to a CMOS Application Specific Integrated Circuit (ASIC) that is solder-bump hybridized, pixel by pixel, to the sensor chip. Each ASIC, which defines a single tile of the detector, consists of 128 × 128 pixels with a pixel size of 150 µm × 150 µm. The full detector used here consisted of six tiles in a 2 × 3 grid with a total of 256 × 384 = 98304 active detector pixels, with gaps between the tiles of 4–6 pixels, depending on position.

Generally, such hybridized pixel array detectors (PADs) are either of the photon-counting or integrating variety (Graafsma, 2010 ▶). Photon-counting detectors process current pulses to identify those that correspond to individual X-ray photons, each of which is then added to digital memory. Dead-time or counting losses occur when the processing times for successive synchrotron pulses, separated by approximately or less than 100 ns, overlap. Because photons arrive stochastically during emission from very short (10–100 ps) bunches, the overlap problem is more severe than often assumed from an average count-rate. By contrast, integrating PADs analogue-integrate the charge arising from the stopped X-rays using a charge-to-voltage amplifier built into each pixel. After an appropriate integration time, the voltage is analogue-to-digital converted and the resultant digital count is read out. Because integration only measures the total charge produced (an electron–hole pair resulting for each 3.65 eV of stopped X-ray energy), extraordinarily high X-ray arrival rates may be accommodated. This is why at X-ray free-electron lasers, where the X-rays arrive in femtoseconds, integrating detectors are used (Graafsma, 2010 ▶). However, the voltage range of the integration amplifier is finite, so only a limited number of photons may be accumulated before the amplifier voltage saturates. This limits the dynamic range per exposure to, generally, hundreds to a few thousands of hard X-rays, far short for the dynamic range required for CDI experiments.

The Mixed-Mode PAD, as its name implies, uses a mode that is a mixture between counting and integrating approaches: charge is accumulated until the output of the front-end integration stage passes a programmed voltage threshold, generally equivalent to several hundred 8-keV photons. When the integration reaches this level, an in-pixel circuit is engaged that removes a fixed amount of charge, typically also set to be equivalent to several hundred 8-keV photons, from the integration amplifier capacitor. This charge-removal process can occur concurrently with the arrival of charge from newly stopped X-rays, *i.e.* the charge-removal process incurs no dead-time. An in-pixel digital counter records the number of times the charge removal circuit is triggered. At the end of the integration period, the in-pixel digital counter is read out, as well as the analog output of the front-end integrating amplifier. The analog output is digitized with off-chip electronics and is combined with the digital counter outputs to measure the total charge produced by X-rays absorbed in the sensor with a resulting full-well exceeding 4 × 10^7^ photons. X-ray images can be read into computer memory at rates exceeding 1000 frames a second. Each pixel can accommodate an average count rate exceeding 10^8^ 8-keV photons pixel^−1^ s^−1^, with instantaneous count rates greater than 10^12^ photons pixel^−1^ s^−1^. Moreover, individual photons are readily seen in the low-fluence parts of the image with a single X-ray signal-to-readout-noise ratio of about 6.

As an example for applications in coherent X-ray diffractive imaging we report, below, on a ptychographic experiment at PETRA III with hard X-rays (7.9 keV) using a weakly attenuated beam with a maximum flux of ∼4.4 × 10^7^ photons pixel^−1^ s^−1^ (2.0 × 10^8^ photons pixel^−1^ s^−1^ in ‘still images’ of the empty beam).

## 2. Experiment   

The experiment was performed at the P10 Coherence Beamline of the PETRA III synchrotron at DESY, Germany. The undulator beam, monochromated to an energy of 7.9 keV by a Si(111) double-crystal monochromator, was focused using the two Kirkpatrick–Baez (KB) mirrors of the Göttingen Instrument for Nano-Imaging with X-rays (GINIX) (Kalbfleisch *et al.*, 2011 ▶). The configuration of the instrument, as used here, has been described previously (Giewekemeyer *et al.*, 2013 ▶). A first pair of slits, located about 84 m downstream of the source, was used to confine the beam to a lateral size of 0.4 mm × 0.4 mm (horizontal × vertical gap width). About 2.9 m further downstream, *i.e.* immediately upstream of the KB mirrors, a laterally coherent fraction of 0.1 mm × 0.1 mm was selected out of the incident wavefield by two pairs of hybrid metal/single-crystal slit blades (Xenocs, France). A pinhole (tungsten, Ø = 10 µm, 20 µm thickness) was placed 8 mm upstream of the (nominal) focal plane in order to define a laterally well confined focus for ptychographic imaging (Giewekemeyer *et al.*, 2013 ▶).

**Figure 1:**
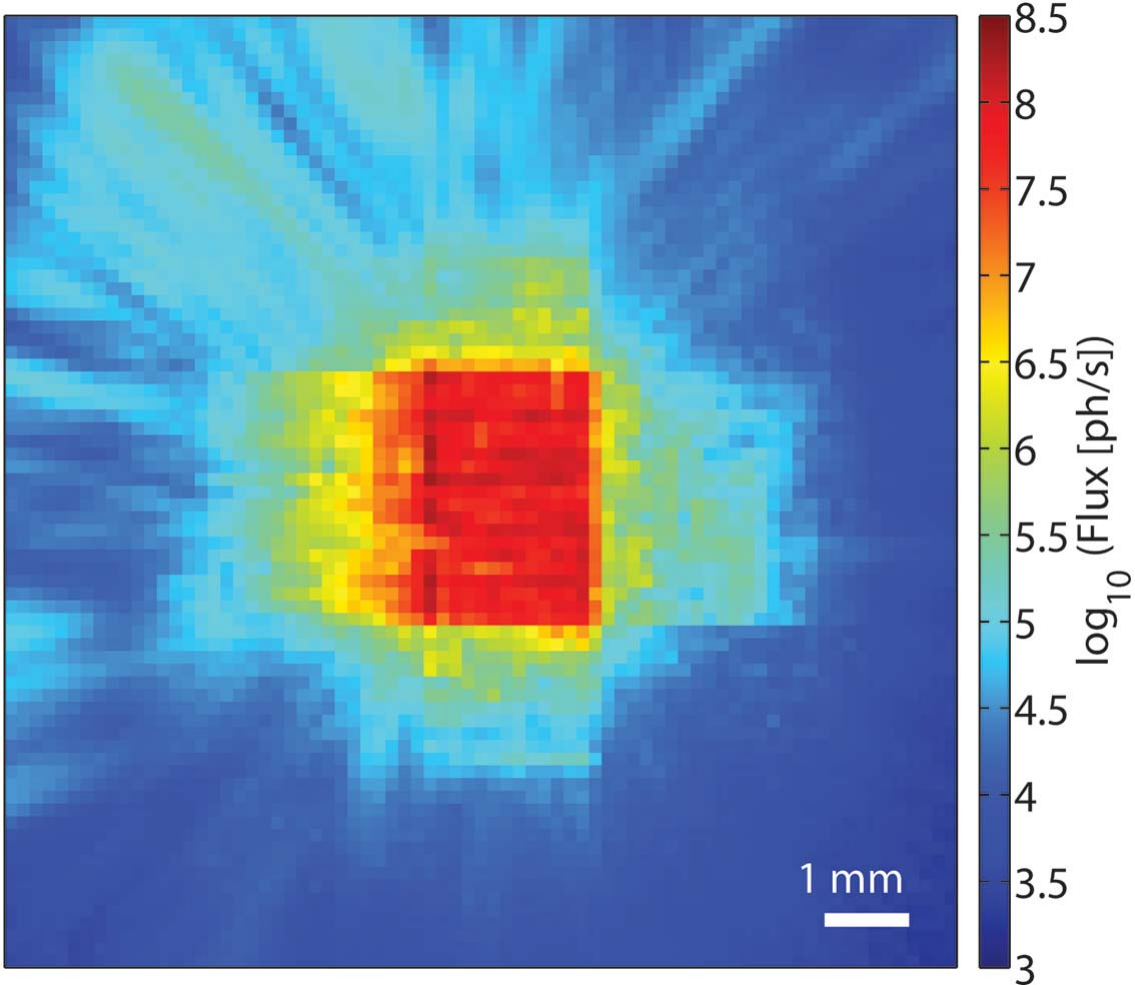
Central region of the KB far-field measured without any attenuators for 0.1 s illumination time. The maximum flux per pixel is 2.0 × 10^8^ photons s^−1^.

The MM-PAD detector was placed approximately 5.07 m downstream of the focal plane, behind an evacuated flight tube covering most of the distance between the focus and the detector. Both the detector and the sample were kept under ambient conditions, not physically connected to the flight tube. For ptychographic imaging, a tantalum Siemens star test structure (model ATN XRESO 50HC, NTT-AT Corporation, Japan) with a nominal structure thickness of 500 nm and finest feature size of 50 nm was mounted onto a piezo scanning stage (Physik Instrumente, Germany). The dataset which is presented here has been obtained by scanning the sample through the focal plane on a Cartesian grid with a lateral step size of 100 nm in both the horizontal and vertical direction, collecting in total 31 × 31 = 961 two-dimensional diffraction patterns on the detector. The illumination time was 0.1 s per frame, resulting in a net illumination time of around 1.6 min. A mild attenuation of a factor of 4.3 was used for this scan. Nevertheless, images without attenuation could be measured as well (see Fig. 1 ▶).

## 3. Analysis   

### 3.1. Detector calibration   

Charge integrating detectors like the MM-PAD require a dark-image subtraction to isolate the X-ray-generated signal from DC offsets in pixels and diode leakage current. Measurement of the dark-image is repeatable and is performed simply by taking images with no incident X-rays (*e.g.*, with the shutter closed). Since any image recorded by the detector has electronic noise associated with it, many dark-images are recorded and averaged to minimize the contribution of offset errors on the measurement of X-ray images. In this experiment, sets of 100 dark-images were periodically collected.

**Figure 2:**
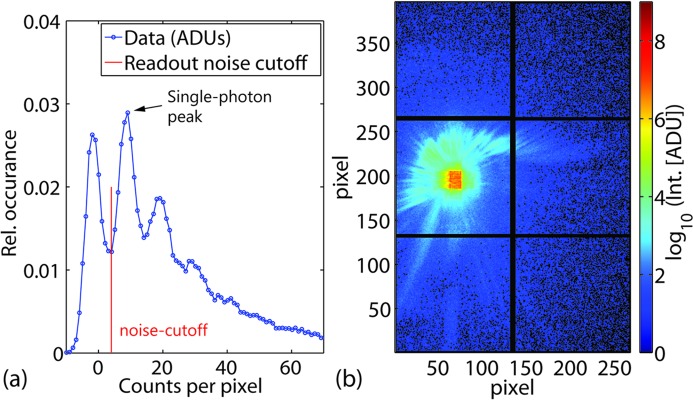
(*a*) Section from a histogram of analog-to-digital units resulting from an empty beam exposure, with many detector pixels illuminated by single photons only. The vertical red line in the histogram plot marks the offset of 4 ADUs that was used to subtract the zero-photon noise. Its peak can be clearly separated from the single-photon peak at 10.8 ADUs. (*b*) Scattering pattern of the empty beam that was used for plotting the histogram on the left. In this image, all values below the threshold of 4 ADUs (red line in subfigure on the left) have been set to 0, corresponding to the black pixels containing no ADUs.

In addition to dark-image subtraction, the 3 × 2 tiled version of the MM-PAD used in this experiment had small frame-to-frame DC offsets in each of the tiles that had to be corrected to achieve the best measurements of the diffraction patterns. The magnitudes of these offsets were on the single-photon level and are thought to originate in the peripheral electronics. The tile-by-tile correction of these offsets was performed by histogramming the pixel values, after dark-image subtraction, of each tile. Since the detector noise is much less than the signal from an 8 keV photon, discrete peaks in the histogrammed signal level can be identified and associated with discrete numbers of photons per pixel. The discrete peaks, for tiles exposed to low fluence, include a peak for zero photons (see Fig. 2 ▶). The position of this peak was determined by fitting a Gaussian to it and using the fitted position of this Gaussian as an offset correction. When the signal in a tile was high and there were not enough pixels with zero photons to give a reliable Gaussian fit, the correction was not performed. In these tiles the magnitude of the correction is generally much less than the Poisson noise associated with the X-rays being measured.

The analog gain across the MM-PAD is known to vary by 0.5% RMS (Green *et al.*, 2013 ▶). This gain variation was not corrected for in the data analysed. For large signals (greater than ∼200 photons pixel^−1^), charge-removal cycles are counted. Since the amount of charge removed in each cycle tracks the analog gain variation, the gain variation for large signals is less than that for small signals.

Owing to the electric field lines diverging at the edge of the sensor, the pixels at the edge of each tile collect charge over a wider area than the inner pixels. This effect has been included in the calibration by application of a correction factor to the measured signal in these pixels. The correction factor was determined empirically by comparison of the signal in the edge pixels to the neighboring inner column or row at a high overall signal level.

For conversion of ADU values into photon count values, a histogram resulting from a single MM-PAD exposure with a considerable single-photon count was analyzed (see Fig. 2 ▶). More specifically, the ADU/photon conversion factor was determined by fitting a Gaussian to the first peak in the Fourier spectrum of the histogram, leading to a value of 10.80 ADU photon^−1^. This value was then used to convert pixel ADU count values into photon count values.

For ptychographic analysis all ADU values below a threshold of 4 ADUs were set to 0 which is safely below the single-photon peak at 10.80 ADUs (see Fig. 2 ▶).

### 3.2. Ptychographic reconstruction   

**Figure 3:**
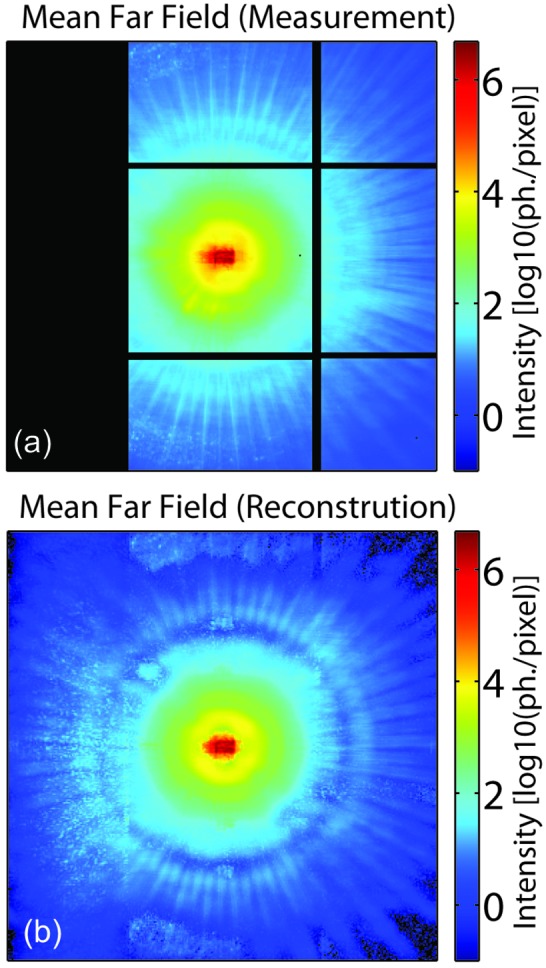
(*a*) Two-dimensional intensity distribution, averaged over all scan points, rescaled into photons per pixel, as measured by the MM-PAD during the ptychographic scan. The black areas correspond to non-sensitive regions, *i.e.* gaps between active sensor areas, invalid pixels, as well as a larger area on the left which is not measured, but which is required to be considered for the high-resolution reconstruction (see main text). (*b*) Two-dimensional intensity distribution, averaged over all scan points, as obtained from the numerically reconstructed exit wavefields.

**Figure 4:**
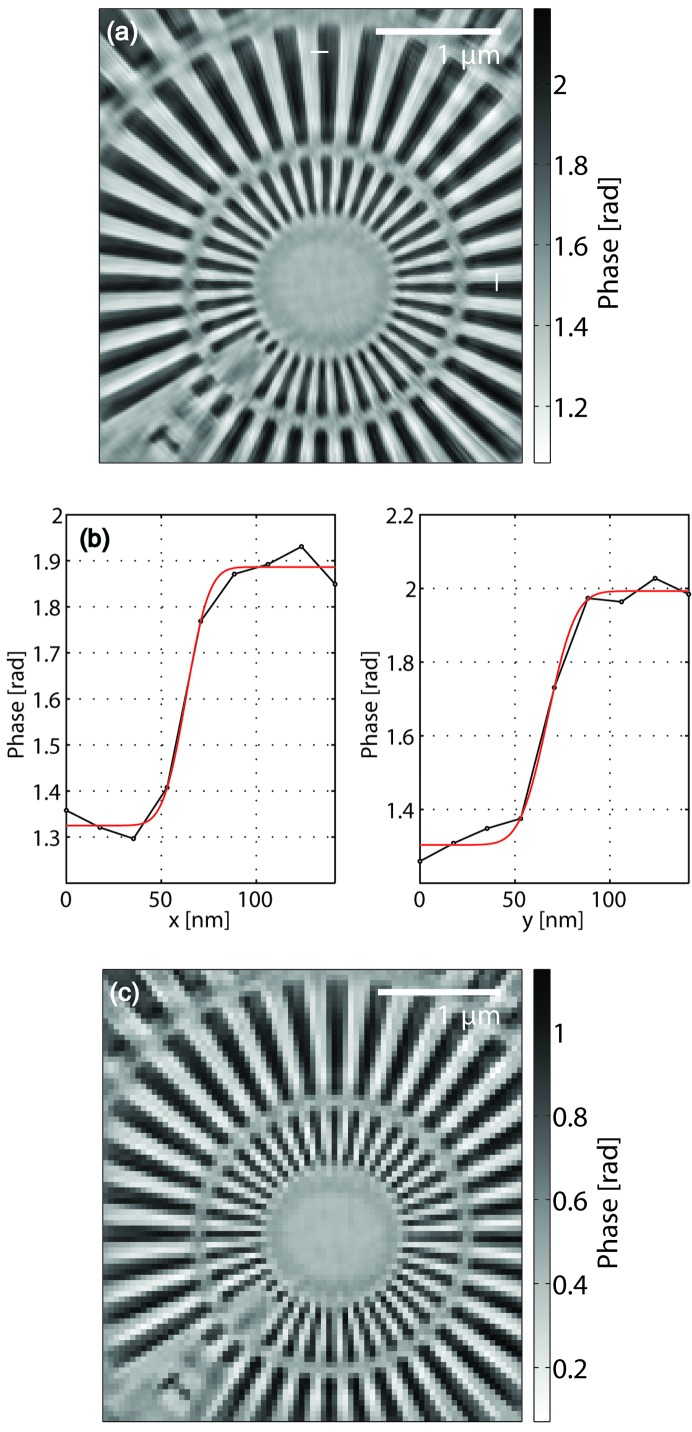
(*a*) Phase reconstruction of the object function (sample) using a detector area of 300 × 300 pixels. Of this area, the leftmost 85 columns have not been measured (see main text and Fig. 3 ▶). For resolution determination, two line scans in the horizontal (*x*) and vertical (*y*) direction are indicated by white lines and plotted as phase *versus* position in (*b*). Here, the red lines indicate the fitted error functions, with corresponding FWHM values of 22 nm in the horizontal direction and 27 nm in the vertical direction. (*c*) Phase reconstruction of the object function using the central 120 × 120 pixels exhibiting the same features as (*a*), but with a resolution limited by the portion of diffraction data used.

The MM-PAD used for this experiment is a 3 × 2 tiling of individual 128 × 128 pixel modules (see Fig. 2*b* ▶). For analysis, a virtual area of 300 × 300 pixels was constructed and MM-PAD data were used to populate the measured subset of the 300 × 300 pixel area. The rest of the area was taken to be equivalent to a non-sensitive ‘detector region’. Fig. 3(*a*) ▶ shows how the data collected were centered into the 300 × 300 virtual pixels for data analysis. This mapping of tiles was determined by using a center of mass formula to identify the (approximate) center of the diffraction pattern.^2^ Since the detector is an asymmetric tiling and the central beam was centered on the left (H) middle (V) tile when recording data, an area 85 pixels wide on the left of the diffraction pattern was not recorded. Because of the strong over-determination of the ptychographic data set and the high scattering signal from the sample, it was possible to reconstruct the diffraction pattern in the non-sensitive portion of the 300 × 300 pixel region used for analysis. This was done by modifying the traditional Fourier update, which leaves insensitive regions unconstrained (Chapman *et al.*, 2006 ▶). In the present analysis, the integrated signal level in the left-hand insensitive 81 columns was constrained to be equal in amplitude to the right-most 81 columns of the diffraction pattern (neglecting here the small area of non-sensitive horizontal gaps between modules). Strictly speaking, such an equality only holds if Friedel symmetry is obeyed; however, we found the present approximation to be experimentally justified in the present case, as shown by the reconstructions presented in Fig. 4 ▶.

The reconstructions were performed by combining a first longer-reconstruction using the Difference Map (DM) algorithm (Thibault *et al.*, 2008 ▶, 2009 ▶) with a subsequent short reconstruction using the extended Ptychographic Iterative Engine (ePIE) (Maiden & Rodenburg, 2009 ▶). The latter used the result of the DM reconstruction as an initial seed for both the illumination function and the object function. When using just the ePIE algorithm alone we found the procedure, for this particular example, often became trapped in local minima which the DM algorithm can escape from effectively. This robustness, however, may lead, especially in the presence of noise, to the possibility of further refinement of the reconstruction, especially after averaging random fluctuations (Thibault & Guizar-Sicairos, 2012 ▶). The observation that ePIE, for this given dataset, performs as a more local optimization scheme may be related to the particular reconstruction parameters explored, but also seems plausible due to the close connection of the ePIE algorithm to a steepest-decent optimization method, a known method of local optimization (Guizar-Sicairos & Fienup, 2008 ▶).

In detail, the DM algorithm was run for 500 iterations, starting with a uniform guess for the object and a two-dimensional Gaussian amplitude (and flat phase) distribution with an intensity FWHM (full width at half-maximum) width of 400 nm × 600 nm for the probe. The refinement of the latter was initiated from the second iteration on. After reaching a steady state, as visible by a flat but random Fourier space error evolution, a mean of the complex object and probe was taken over the last 100 iterations to average out fluctuations in the reconstruction and to increase the reliability of the result (Chapman *et al.*, 2006 ▶; Thibault *et al.*, 2008 ▶). The resulting complex object and illumination were then used as an initial guess for 150 iterations of the ePIE algorithm, averaging over the last 20 iterations to reach the final result (again, starting the probe refinement at iteration 2). The feedback parameters α and β for the ePIE algorithm (Maiden & Rodenburg, 2009 ▶) were both set to 0.95. For both algorithms, the probe was confined by multiplication of a circular mask (Giewekemeyer *et al.*, 2011 ▶) after each probe refinement step. This radially symmetric mask was of a sigmoidal form corresponding to a Butterworth filter function (Gonzalez *et al.*, 2004 ▶) of the form *f*(*r*) = 1/[1+ (*r*/*r*_0_)^2*n*^] with *n* = 10 and *r*_0_ = 1.1*D*/2 where *D* is the diameter of the numerical field of view. After the final ePIE reconstruction, a residual linear phase ramp was removed from the object, largely following the procedure described by Guizar-Sicairos *et al.* (2011 ▶) with tools described by Guizar-Sicairos *et al.* (2008 ▶).

## 4. Results and discussion   

### 4.1. Ptychographic reconstruction   

**Figure 5:**
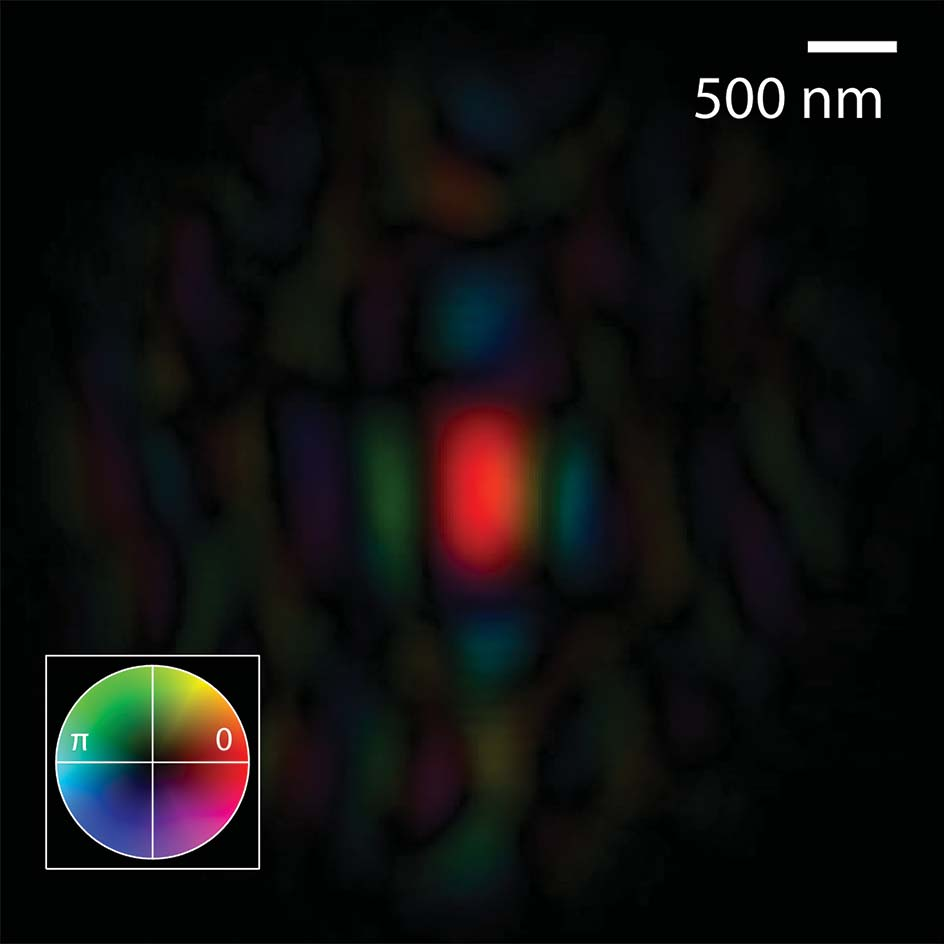
Complex wavefield reconstructed from the high-resolution dataset, numerically propagated into the focal plane. Phase is decoded in color, amplitude in brightness, as per the colorwheel on the lower left.

The reconstruction of the complex illumination function is shown in Fig. 5 ▶. The result strongly resembles the reconstruction from a measurement with very similar geometrical settings obtained about one year earlier, using strong attenuation and the Pilatus detector (Giewekemeyer *et al.*, 2013 ▶). This illustrates the stability of the GINIX set-up as well as the reliability of the reconstruction method. The key difference here is the use of strongly increased flux in the present experiment, made possible by the use of a detector with a higher dynamic range.

**Figure 6:**
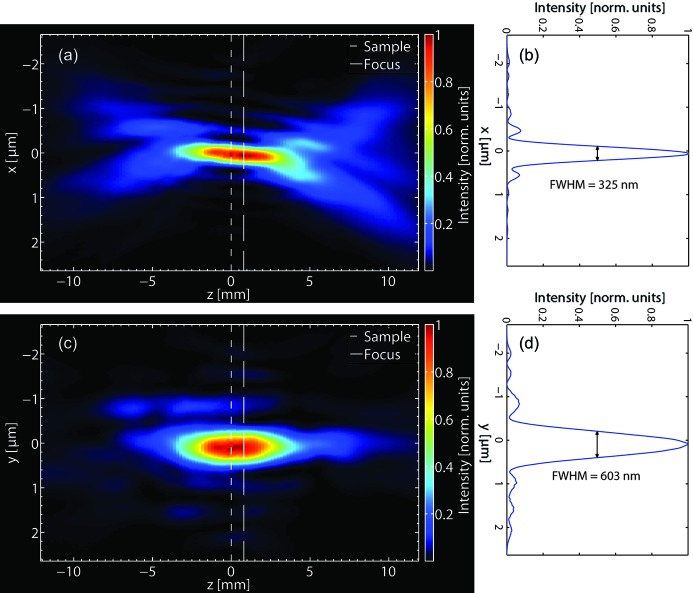
Reconstructed intensity distribution near the focus (*a*) in the sagittal (*xz*) and (*c*) in the meridional (*yz*) plane, where the optical axis is oriented in the *z*-direction. (*b*) and (*d*) show corresponding line cuts through the intensity in the focal plane.

Numerically propagating the determined complex wavefield back and forth by a few millimeters yields a three-dimensional view of the wavefield in the vicinity of the focus (see Fig. 6 ▶). Even though the position of the focal plane, as determined by an intensity-summation criterion (Guizar-Sicairos, 2010 ▶), differs from that of the sample plane by about 1 mm, the sample was placed still well within the depth of the focus with an intensity FWHM of approximately 325 nm in the horizontal and 603 nm in the vertical direction. The maximum reconstructed intensity in a single pixel within the focal plane as determined from the reconstruction was of the order of 1 × 10^10^ photons µm^−2^ s^−1^.

The corresponding phase reconstruction of the object is shown in Fig. 4(*a*) ▶. While in some areas (such as in the lower-left and upper-right region) some imaging artifacts can be observed (which may be attributed to stage drift and a remaining finite degree of partial coherence) there are features in the reconstruction which are imaged sharply down to nearly the real-space pixel size. Gaussian error function fits to line scans in the horizontal and vertical direction yield corresponding FWHM values of 22 nm and 27 nm, respectively (see Fig. 4*b* ▶). The increment in the phase in the horizontal and vertical line profiles is 0.56 and 0.69 in the fitted curves, and 0.63 and 0.77 in the largest difference of considered pixel values, compared to a reference value of 0.757 rad phase difference of the 500 nm tantalum material at 7.9 keV, as measured in an earlier experiment with high attenuation (Wilke *et al.*, 2013 ▶).^3^

In addition, we also show an object reconstruction resulting from a dataset which only takes into account the central 120 × 120 detector pixels, *i.e.* without the need to incorporate additional non-measured regions (see Fig. 4*c* ▶). It can be seen that the remaining artifacts are very similar in both images, indicating a successful reconstruction of the unknown detector regions.

This is further supported by comparison of the measured diffraction data, averaged over all scan points, with the mean of the reconstructed far-field diffraction patterns (see Fig. 3 ▶). The reconstructed far-field shown here resembles so-called ‘super resolution’ reconstructions that have been examined before for ptychographic CDI and demonstrated for the case of optical wavelengths (Maiden *et al.*, 2011 ▶).

### 4.2. Detector performance   

As visible in Fig. 2(*a*) ▶, the single-photon peak can clearly be separated from the zero-photon noise, giving the detector a reliable single-photon-counting capability at the used X-ray energy of 7.9 keV.

Additionally, it was possible to measure the far-field of the unattenuated KB beam, as shown in Fig. 1 ▶ where the central region of the empty beam scattering pattern is shown, originating from an exposure of 0.1 s. We observed no saturation in this image, with a maximum flux of 2.0 × 10^8^ photons pixel^−1^ s^−1^. According to the total counts on the MM-PAD, the (coherent) flux in the KB beam was 2.25 × 10^10^ photons s^−1^. In contrast to the ptychographic scan, here the beam-defining slits were set to 0.1 mm × 0.2 mm.

## 5. Conclusions   

In summary, we have used (far-field) X-ray coherent diffractive imaging, a technique which is inherently demanding on detector performance, particularly its dynamic range, to demonstrate the capabilities of the MM-PAD, a novel high-dynamic-range detector that is based on a combined integrating and counting detection principle.

It has been shown that the MM-PAD is capable of reliable single-photon detection at 7.9 keV and, at the same time, of measuring the far-field of an unattenuated undulator beam, focused by a pair of two KB mirrors. The flux values determined here exceed 1 × 10^8^ photons pixel^−1^ s^−1^, beyond values currently achieved by photon-counting detectors (Trueb *et al.*, 2012 ▶).

As an example for applications in CDI, a ptychographic reconstruction of the KB wavefield was performed, applying a maximum intensity of about 1 × 10^10^ photons µm^−2^ s^−1^ within a focus with a size of ∼325 nm × 603 nm (FWHM). Due to the high dynamic range of the detector, this measurement was possible without using a (semi-transparent) beamstop (Wilke *et al.*, 2013 ▶) or a phase diffusor for amplification of high spatial frequencies in the diffraction pattern (Maiden *et al.*, 2013 ▶), both processes that complicate the analysis of CDI data.

Owing to its demonstrated performance, the MM-PAD opens up a wide range of CDI experiments requiring both a relatively large probe size, in the range of several hundred nanometers, and a high maximum fluence in the range of 10^10^ photons µm^−2^.

Owing to the non-square tile arrangement of the MM-PAD prototype (2 × 3 tiles) used in this work, a centro-symmetric placement of the far-field pattern onto the detector was not possible. As indicated in earlier works on super-resolution effects in ptychography (Maiden *et al.*, 2011 ▶), it was, however, possible to use a centro-symmetric numerical region of interest of the far-field pattern, of which a considerable fraction could be successfully recovered by the ptychographic reconstruction process. Ideally, a future detector model optimized for CDI applications could use a symmetric tiled configuration of the MM-PAD, *e.g.* in a 3 × 3, or larger, arrangement. Such larger format tilings with less space between the tiles are perfectly feasible and are limited only by development time and money.

## Footnotes

1 The MM-PAD concept was developed collaboratively by our detector group at Cornell University and Area Detector Systems Corporation (ADSC) in Poway, CA, USA. The small detector used for the experiments described in this article uses the chips that resulted from the collaboration, configured into a small detector of 2 × 3 tiles designed by the Cornell group.

2 For the use of such a procedure, in a different context, see, for example, Berenguer *et al.* (2014 ▶).

3 The theoretically expected value is 0.83 rad. This, however, assumes a homogeneous thickness of 500 nm throughout the sample and tantalum bulk density.
